# Appropriate time for ejection fraction reassessment after revascularization in patients with left ventricular dysfunction for risk stratification of sudden cardiac death

**DOI:** 10.1002/clc.24162

**Published:** 2023-11-07

**Authors:** Shaoping Wang, Yi Lyu, Shujuan Cheng, Zheng Wu, Shiying Li, Ze Zheng, Xiaoyan Gu, Jinhua Li, Jinghua Liu, Bijan J. Borah

**Affiliations:** ^1^ Department of Cardiology, Beijing Anzhen Hospital, Beijing Institute of Heart Lung and Blood Vessel Diseases Capital Medical University Beijing China; ^2^ Department of Health Sciences Research Mayo Clinic Rochester Minnesota USA; ^3^ Department of Anesthesiology, Minhang Hospital Fudan University Shanghai China; ^4^ Department of Echocardiography, Beijing Anzhen Hospital, Beijing Institute of Heart Lung and Blood Vessel Diseases Capital Medical University Beijing China; ^5^ Department of Cardiovascular Surgery, Beijing Anzhen Hospital, Beijing Institute of Heart Lung and Blood Vessel Diseases Capital Medical University Beijing China; ^6^ Robert D. and Patricia E. Kern Center for Science of Health Care Delivery Mayo Clinic Rochester Minnesota USA

**Keywords:** ejection fraction, implantable cardioverter defibrillator, revascularization, sudden cardiac death

## Abstract

**Background:**

Appropriate time for ejection fraction (EF) reassessment after revascularization in patients with left ventricular dysfunction has not been investigated comprehensively, although 3 months after revascularization is recommended to stratify the risk of sudden cardiac death (SCD).

**Hypothesis:**

EF reassessed within different timeframe after revascularization may have incosistent contribution for risk stratification of SCD.

**Methods:**

Patients who had EF ≤ 40% before revascularization and had EF reassessment at least once during follow‐up were included. The role of early (<3 months) versus late (3–12 months) EF measurements in prediction of all‐cause mortality and SCD were compared.

**Results:**

A total of 1589 patients were identified. EF reassessed <3 months was lower than EF reassessed within 3–12 months (42.1 ± 9.7% vs. 45.8 ± 10.8%; *p* < .01). Among 1069 patients who had EF reassessed <3 months, EF ≤ 35% was associated with a higher risk of all‐cause mortality (hazard ratio [HR], 1.67; 95% confidence interval [CI], 1.22–2.29; *p* < .01), but had no association with the risk of SCD (HR, 1.44; 95% CI, 0.84–2.48; *p* = .18). By contrast, among 595 patients who had EF reassessed within 3–12 months, EF ≤ 35% was associated with higher risks of both all‐cause death (HR, 1.81; 95% CI, 1.06–3.10; *p* = .03) and SCD (HR, 2.71; 95% CI, 1.31–5.61; *p* < .01). The relative contribution of SCD to all‐cause death was higher in patients with EF ≤ 35% than patients with EF > 35% when EF was reassessed within 3–12 months (*p* = .04). However, when EF was reassessed <3 months, the mode of death was similar in patients with EF ≤ 35% versus >35% (*p* = .85).

**Conclusions:**

3 to 12 months after revascularization may be appropriate for cardiac function reassessment and SCD risk stratification.

## Graphical abstract

Ejection fraction (EF) changed over time after revascularization in patients with ischemic cardiomyopathy. When EF was reassessed early (<3 months) after revascularization, EF ≤ 35% predicted the risk of all‐cause death but not sudden cardiac death (SCD). By contrast, when EF was reassessed within 3–12 months, EF ≤ 35% was associated with higher risks of both SCD and all‐cause death.

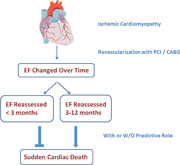

## 1 INTRODUCTION

Ejection fraction (EF) has already been accepted as a critical factor for risk stratification for sudden cardiac death (SCD), and one of the criteria for placement of implantable cardioverter defibrillator (ICD) for primary or secondary SCD prevention.^1^, ^2^ However, the extent of EF recovery after revascularization in patients with coronary artery disease (CAD) and preoperative left ventricular (LV) dysfunction is obscured.^3^, ^4^, ^5^, ^6^, ^7^, ^8^, ^9^, ^10^, ^11^ Persistent LV dysfunction with EF ≤ 35% after coronary artery bypass graft (CABG) has been reported ranging from 25% to 74%.^7^, ^8^ One explanation is the difference in baseline characteristics of patient cohorts, such as the myocardial viability^4^, ^6^ and preoperative EF.^10^ Another potential explanation that has not yet been addressed clearly is the timing of EF reassessment following revascularization. The reassessment time for EF comparison was diverse from predischarge^5^ to 24 months^10^ after revascularization. The EF might change over time after revascularization in patients with LV dysfunction.^5^, ^9^

Current guideline recommends that patients with LV dysfunction undergo a reassessment of EF 3 months after revascularization to evaluate the necessity for ICD placement.^12^, ^13^ The rationale for waiting 90 days after revascularization is based upon the premise that LV function can be improved sufficiently to raise the EF to above 35% and the clinical trials^14^, ^15^ which enrolled patients with a 3‐month waiting period after CABG before the implantation of an ICD. However, this time point for EF reassessment is not supported by solid evidence, as the guideline document^13^ said “the 3‐month term was chosen because it was used in some randomized clinical trials related to timing for device implantation,” “the waiting period of 3 months after diagnosis of a new cardiomyopathy or revascularization procedure is arbitrary.”

Yet, some trials^16^, ^17^, ^18^ recorded EF measurement before the completion of 3 months after revascularization and had ICD implanted in patients with a higher risk of ventricular arrhythmia. However, the trials failed to indicate a clear benefit of ICD in overall mortality reduction. The potential reasons were controversial.^19^, ^20^ As revascularization may have an important time‐dependent effect on EF evolution, we hypothesize that EF measurement 3 months earlier after revascularization does not predict the risk of SCD.

This study aimed to investigate (1) the change of EF over time after revascularization; (2) association between EF reassessed <3 months and the risk of long‐term mortality and SCD; (3) association between EF reassessed within 3–12 months and the risk of long‐term mortality and SCD; (4) change in the composition of mortality risk (SCD vs. non‐SCD deaths) stratified by EF reassessed <3 months and within 3–12 months.

## 2 MATERIAL AND METHODS

### 2.1 Patient selection

This was a real‐world cohort study that used data from Beijing Anzhen Hospital. The study was registered in the Chinese Clinical Trial Registry (No. ChiCTR2100044378). The study protocol was approved by the hospital's ethics committee. Patients with initial EF ≤ 40% who underwent either percutaneous coronary intervention (PCI) with drug‐eluting stent or CABG due to CAD between January 2005 to December 2014 were screened. If patients had multiple PCI or CABG during the follow‐up, the first qualifying procedure was used. Patients who had EF reassessment at least once after revascularization were enrolled.

### 2.2 Data collection and definitions

Baseline characteristics including demographics, comorbidities, EF, angiographic results, and medicines at discharge were captured from patients’ medical records. Left main disease was defined as at least 50% diameter stenosis in the left main vessel. Multivessel disease was defined as the presence of coronary luminal diameter stenosis ≥70% in more than one of the three major epicardial vessels. Complete revascularization was defined as successful PCI of all angiographically significant lesions (≥70% diameter stenosis) in three coronary arteries and their major branches. For CABG procedures, grafting to every primary coronary artery with ≥70% diameter stenosis was accepted as complete revascularization.

All EF measurements were assessed by echocardiography in Beijing Anzhen Hospital. The initial EF was defined as being measured within 30 days before the index PCI or CABG. During the follow‐up, EF values were recorded and separated into the following time intervals: <3, 3–6, 6–9, 9–12, and the last measurement (>12 months). For patients who had multiple echocardiography measurements within each time interval of <3, 3–6, 6–9, and 9–12 months, the first available measurement was used. For patients with multiple measurements in the last interval (>12 months), the last available measurement was used. For patients defined as having EF reassessed within 3–12 months, the first available measurement was used if the patients had multiple measurements within 3–12 months.

### 2.3 Outcomes

Outcome data were obtained from both medical records at Beijing Anzhen Hospital as well as through phone contact. Death was categorized as cardiac and noncardiac death. Cardiac death was further categorized SCD and non‐SCD.^21^ Death with insufficient information to make a reasonable decision as to the cause of death was classified as death due to unknown causes. According to a modified Hinkle–Thaler system,^22^ SCD was defined as a sudden, unexpected cardiac death, which included those who: (1) died suddenly and unexpectedly within 1 h of cardiac symptoms in the absence of progressive cardiac deterioration; (2) died unexpectedly in bed during sleep; and (3) died unexpectedly within 24 h after last being seen alive.

### 2.4 Statistical analysis

Categorical variables were summarized as frequencies, with percentages and continuous variables were expressed as mean ± SD. Comparison of EF at each time period was by one‐way ANOVA followed by Bonferroni multiple‐comparison tests. A repeated measures mixed model was used to analyze changes in EF over time between the PCI and CABG groups.

Patient characteristics among cohorts with EF reassessment at least once, with EF reassessed <3 months and with EF reassessed within 3–12 months were compared by one‐way ANOVA for continuous variables and chi‐square test for categorical variables.

Patients (*n* = 1069) who had EF reassessed <3 months were included in the analysis of the association between EF reassessed <3 months and the risk of all‐cause mortality and SCD. Correspondingly, patients (*n* = 595) who had EF reassessed within 3–12 months were included in the analysis of the association between EF reassessed within 3–12 months and the risk of all‐cause death and SCD. The follow‐up for these patients started on the day of their EF reassessment. Patients in these two cohorts were all categorized as their reassessed EF being of ≤35% or >35%.^12^, ^13^ Cumulative incidences were estimated by the Kaplan–Meier method and compared by log‐rank test. The risk of all‐cause death was analyzed using a Cox proportional hazard regression model. To identify factors associated with the risk of SCD, a Cox proportional hazard model by treating deaths from other causes as a competing risk was used.^23^ All variables that had marginal association in univariate analysis (*p* < .10) were adopted as independent variables in the multivariate model. For study purposes only, EF reassessed <3 or 3–12 months after revascularization was locked in the model and was not subjected to the selection criteria.

All statistical analyses were based on two‐tailed tests. Values of *p* < .05 were considered statistically significant. Statistical analyses were performed with Stata version 14.0 (College Station).

## 3 RESULTS

### 3.1 Improvement of EF over time

In total, 2852 patients had preoperative EF ≤ 40% who underwent either PCI or CABG. Patients who had concomitant non‐CABG surgery (*n* = 306), and who died in hospital during the index procedure (*n* = 104), and who were lost to follow‐up (*n* = 229), and who had no EF reassessment after revascularization (*n* = 624) were excluded. A total of 1589 patients were finally included in the study, of whom 632 (39.8%) underwent PCI and 957 (60.2%) underwent CABG (Table 1). All the patients had EF reassessed at least once during the follow‐up. The mean age was 65.6 years with a mean initial EF of 35.9%.

**Table 1:** Baseline characteristics.

Patient characteristics	With EF reassessed (*N* = 1589)	With EF reassessed <3 months (*N* = 1069)	With EF reassessed 3–12 months (*N* = 595)	*p* Value
Demographics and history				
Age (years)	65.6 ± 10.5	65.7 ± 10.0	64.0 ± 10.3	<.01
Male sex, *n* (%)	1338 (84.2)	905 (84.7)	498 (83.7)	.87
Weight (kg)	71.6 ± 11.1	71.4 ± 10.9	72.1 ± 10.6	.43
Current smoker, *n* (%)	544 (34.2)	372 (34.8)	213 (35.8)	.79
Hypertension, *n* (%)	831 (52.3)	540 (50.5)	325 (54.6)	.27
Diabetes, *n* (%)	554 (34.9)	374 (35.0)	218 (36.6)	.73
eGFR (mL/min/1.73 m^2^)	84.9 ± 25.3	85.4 ± 26.2	85.8 ± 23.0	.74
Cerebral vascular disease, *n* (%)	142 (8.9)	104 (9.7)	58 (9.8)	.74
Atrial fibrillation, *n* (%)	74 (4.7)	53 (5.0)	26 (4.4)	.86
Bundle branch block	90 (5.7)	66 (6.2)	30 (5.0)	.63
History of MI, *n* (%)	776 (48.8)	520 (48.6)	248 (41.7)	.01
History of anterior MI, *n* (%)	298 (18.8)	160 (15.0)	100 (16.8)	.04
History of PCI, *n* (%)	248 (15.6)	146 (13.7)	88 (14.8)	.38
History of CABG, *n* (%)	31 (2.0)	11 (1.0)	14 (2.35)	.09
Ejection fraction				
Initial EF (%)	35.9 ± 4.6	35.7 ± 4.7	36.4 ± 4.3	.02
Indication for revascularization				
STEMI, *n* (%)	373 (23.5)	243 (22.7)	173 (29.1)	.01
NSTEMI, *n* (%)	127 (8.0)	67 (6.3)	65 (10.9)	<.01
Unstable angina, *n* (%)	496 (31.2)	332 (31.1)	138 (23.2)	<.01
Acute coronary syndrome, *n* (%)	996 (62.7)	642 (60.1)	376 (63.2)	.31
Stable angina, *n* (%)	593 (37.3)	427 (39.9)	219 (36.8)	.31
Angiography and therapy				
Multivessel disease, *n* (%)	1310 (83.5)	931 (87.8)	473 (79.9)	<.01
Left main disease, *n* (%)	124 (7.9)	97 (9.2)	39 (6.6)	.18
PCI treatment, *n* (%)	632 (39.8)	253 (23.7)	304 (51.1)	<.01
CABG treatment, *n* (%)	957 (60.2)	816 (76.3)	291 (48.9)	<.01
Complete revascularization, *n* (%)	919 (58.6)	662 (62.5)	332 (56.1)	.03
ICD/CRT therapy, *n* (%)	12 (0.9)	7 (0.8)	3 (0.6)	.78
Beta blockers	1246 (78.5)	817 (76.6)	486 (81.7)	.05
ACEI/ARB	682 (43.0)	366 (34.3)	274 (46.1)	<.01
Aldosterone blockers	238 (15.0)	133 (12.5)	126 (21.2)	<.01

*Note*: Continuous variables were described using means and SDs. Categorical variables were summarized with their frequencies and corresponding proportions expressed as percentages.

Abbreviations: ACEI, angiotensin‐converting enzyme inhibitor; ARB, angiotensin receptor blocker; CABG, coronary artery bypass graft; CRT, cardiac resynchronization therapy; EF, ejection fraction; eGFR, estimated glomerular filtration rate; ICD, implantable cardioverter defibrillator; MI, myocardial infarction; mo, months; NSTEMI, non‐ST segment elevation myocardial infarction; PCI, percutaneous coronary intervention; STEMI, ST‐segment elevation myocardial infarction.

**Figure 1:**
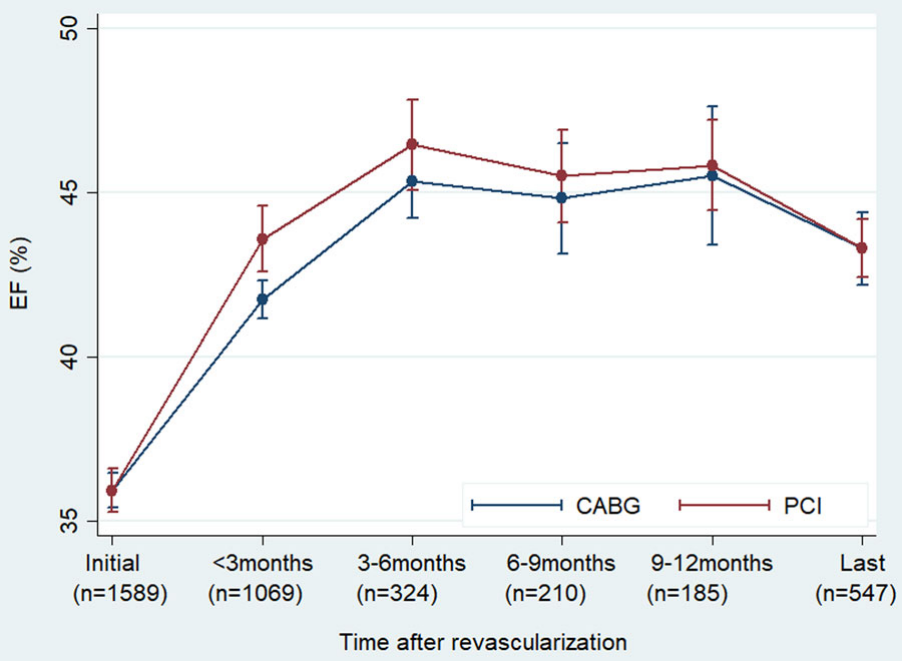
Ejection fraction (EF) improvement over time after revascularization by either coronary bypass grafting (CABG) or percutaneous coronary intervention (PCI). The error bar indicated 95% confidence intervals.

Compared to the initial EF, the EFs at each time interval after revascularization were improved significantly, including the EF reassessed <3 months (PCI 43.7 ± 9.8% vs. 35.9 ± 4.7%, *p* < .01; CABG 41.6 ± 9.6% vs. 35.9 ± 4.5%, *p* < .01) (Figure 1). EF reassessed <3 months in the PCI group was higher than that in the CABG group (*p* = .01). However, in the later time intervals, EF in the PCI group was similar to that in the CABG group. EF improvement peaked at the time of 3–6 months and persisted until 9–12 months. EFs reassessed at 3–6 months (46.3 ± 10.6%), 6–9 months (45.5 ± 11.0%), and 9–12 months (46.0 ± 10.8%) were statistically similar and all signifiant higher than that at <3 months (42.1 ± 9.7%, *p* < .01 for all). The last EF measurement (43.5 ± 11.6%) was reassessed 4.1 ± 2.4 years after revascularization and was found to significantly decrease in comparison to each measurement at 3–6 months (*p* < .01), 6–9 months (*p* = .04), and 9–12 months (*p* = .01).

### 3.2 Associations between EF reassessed <3 months and outcomes

**Figure 2:**
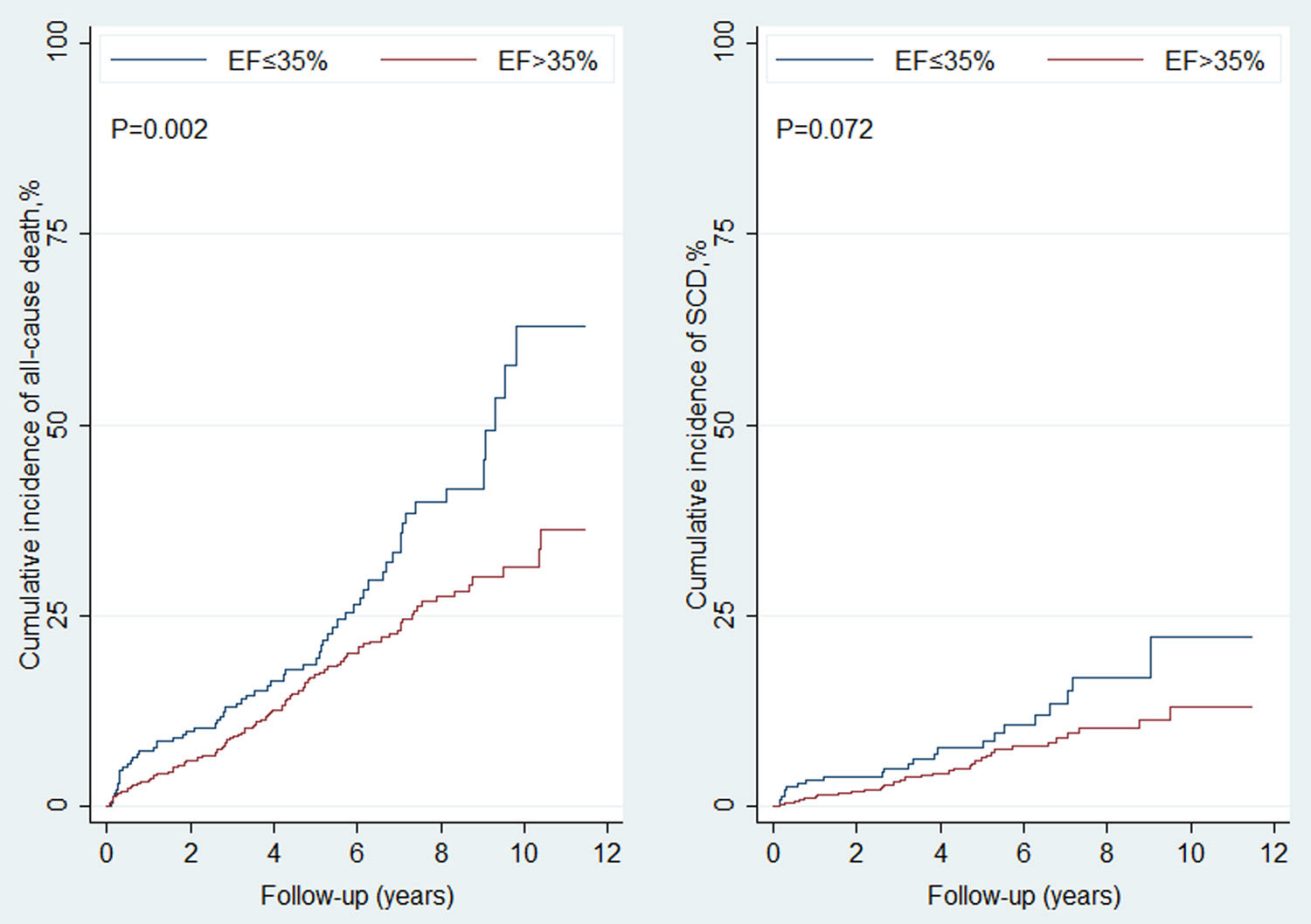
Ejection fraction (EF) ≤ 35% versus EF > 35% for risks of all‐cause death (left) and sudden cardiac death (right). EF was reassessed <3 months after revascularization.

In total, 1069 patients had EF reassessed <3 months after revascularization (Table 1). After a mean follow‐up of 4.0 years, there were 181 (16.9%) deaths, which included 63 (5.9%) SCDs. A total of 276 (25.9%) patients were with reassessed EF ≤ 35% and the EF value was 30.8 ± 4.3%. Mean EF in the patient cohort having reassessed EF > 35% was 46.1 ± 7.7%. EF ≤ 35% was associated with a higher risk of all‐cause mortality (hazard ratio [HR], 1.67; 95% confidence interval [CI], 1.22–2.29; *p* = .002) (Supporting Information: Table 1 and Figure 2). However, it had no significant association with SCD risk (HR, 1.44; 95% CI, 0.84–2.48; *p* = .181). Old age (HR, 1.03; 95% CI, 1.01–1.05; *p* < .001) was also associated with higher risk of all‐cause death.

### 3.3 Associations between EF reassessed 3–12 months and outcomes

**Figure 3:**
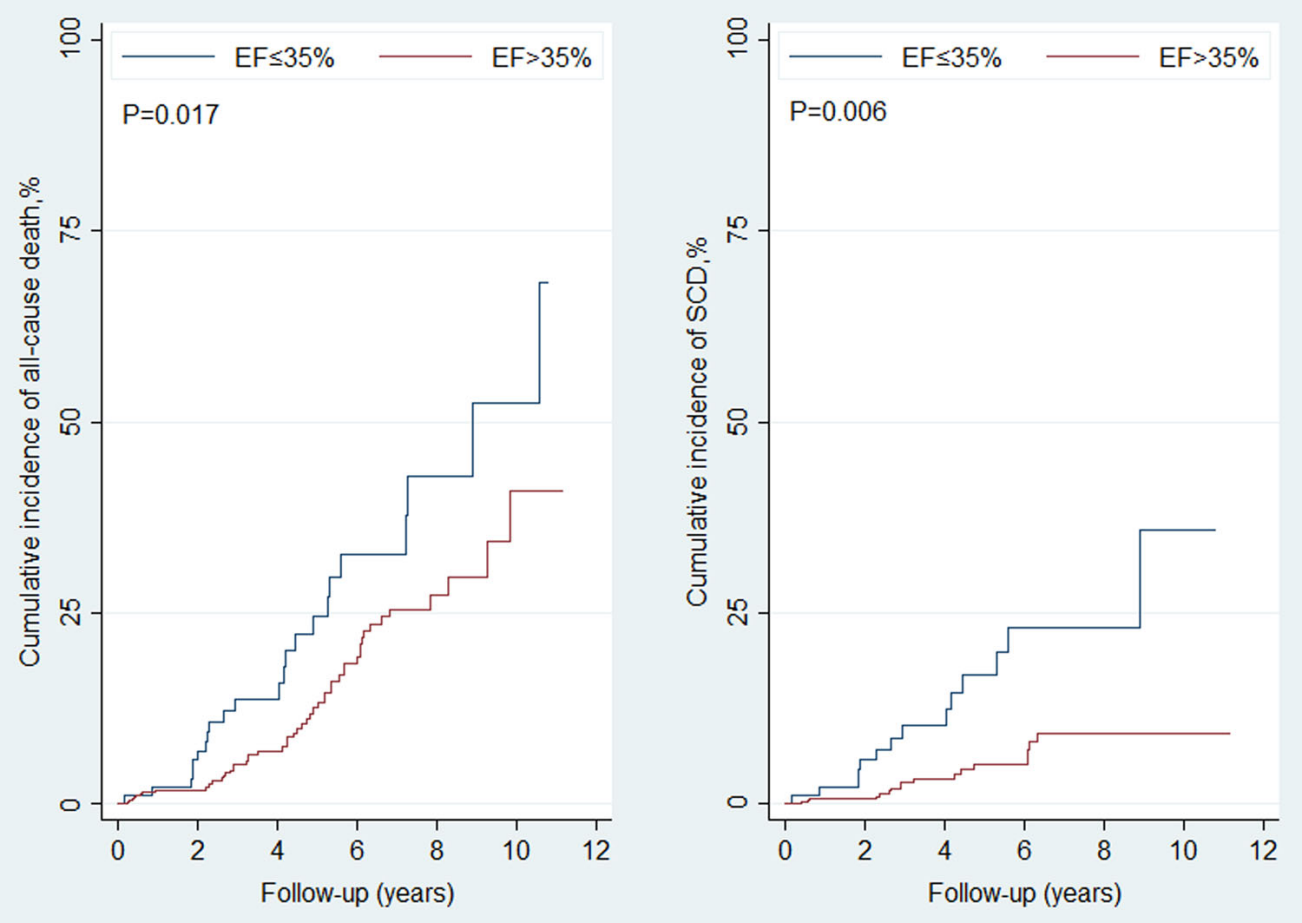
Ejection fraction (EF) ≤ 35% versus EF > 35% for risk of all‐cause death (left) and sudden cardiac death (right). EF was reassessed within 3–12 months after revascularization.

In total, 595 patients (Table 1) had EF reassessed within 3–12 months after revascularization and were followed for a mean duration of 3.6 years. There were 75 (12.6%) deaths, which included 31 (5.2%) SCDs. A total of 107 (18.0%) patients had reassessed EF ≤ 35%. The mean EF was 30.7 ± 3.8% in group of EF ≤ 35% and was 49.1 ± 8.8% in group of EF > 35%. EF ≤ 35% was associated with a higher risk of both all‐cause death (HR, 1.81; 95% CI, 1.06–3.10; *p* = .029) and SCD (HR, 2.71; 95% CI, 1.31–5.61; *p* = .007) (Supporting Information: Table 2 and Figure 3). Higher preoperative eGFR was another independent correlate which associated with lower risk of all‐cause death (HR, 0.98; 95% CI, 0.97–0.99; *p* < .001).

### 3.4 Change of causes of death

**Figure 4:**
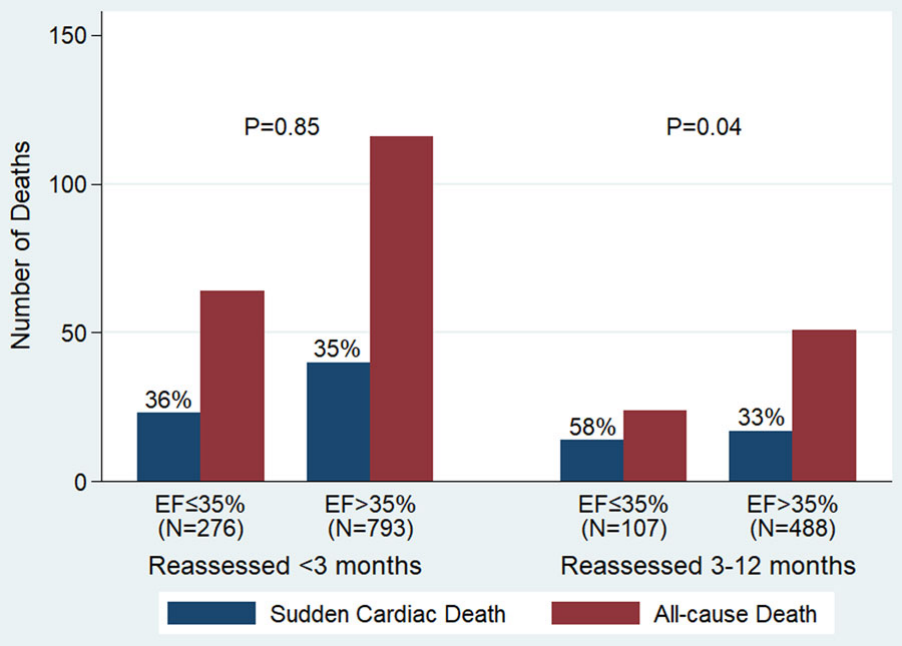
Relation between time of ejection fraction (EF) reassessment and mode of death, subdivided by EF ≤35% or >35%.

Among patients with EF reassessed <3 months, the percentage of overall death accounted for SCD was similar regardless of whether the EF was ≤35% or >35% (36% vs. 35%, *p* = .85) (Figure 4). However, when the EF was reassessed within 3–12 months after revascularization, 58% of deaths were SCD in group with EF ≤35%, whereas only 33% of deaths were SCD in group of EF >35% (*p* = .04).

## 4 DISCUSSION

In the present study of CAD patients with reduced EF (≤40%), we found that (1) EF after revascularization changed over time. However, EF reassessment taken within 3–12 months remained relatively stable indicating that this time period may be more appropriate for evaluating cardiac function recovery; (2) EF reassessed within 3–12 months after revascularization had a predictive role for the risk of SCD but not EF reassessed <3 months; (3) EF reassessed within 3–12 months influenced the cause of death but not EF reassessed <3 months.

Evolution of EF over time after myocardial revascularization in patients with LV dysfunction has not yet been addressed comprehensively. In the present study, all patients had initial EF ≤ 40% and underwent either PCI or CABG. There was no statistically significant difference in EF reassessed among the time intervals of 3–6, 6–9, and 9–12 months. However, EF reassessed <3 months after revascularization was significantly less than EF reassessed during each of the above three time intervals.

According to the EF assessed <3 months, patients who had EF ≤ 35% had a similar risk of SCD compared to patients with EF > 35%, though EF ≤ 35% was associated with a higher risk of mortality. This implied that EF ≤ 35% as a traditional risk factor of SCD, when it was reassessed <3 months, may not be predictive enough to discriminate high‐risk population that was prone to suffer SCD. Moreover, it did not appear to influence the cause of death (SCD vs. all‐cause). Thus, the survival benefit of ICD therapy on this patient cohort with EF ≤ 35% would be lacking because the proportion of patients who benefit from ICD is less. In the trials of DINAMIT^17^ and IRIS,^18^ according to the EF assessed <3 months, ICD therapy was associated with a reduction in the rate of death due to arrhythmia, but did not reduce overall mortality in patients with recent MI. Our data provided another potential explanation and supported the rationale for waiting 90 days after revascularization to a decision making of ICD therapy.

Substudies of primary prevention trials showed that ICD therapy had an increasing survival benefit as time from revascularization increased.^24^, ^25^, ^26^ In a substudy of MADIT‐II,^24^ 951 patients with prior coronary revascularization were investigated. ICD benefited only patients enrolled for at least 6 months after revascularization. In a retrospective study of patients with severe LV dysfunction,^8^ all patients underwent both CABG and early ICD implantation. The mean duration between CABG and ICD implantation was 15 ± 22 days. In all, 74% patients remained EF ≤ 35% after CABG. EF ≤ 35% was associated with a higher risk of total death, but failed to predict the outcome of appropriate ICD therapy. Our data indicated that the decision of ICD based on the EF obtained in the early phase after revascularization might be rendered problematic. Three to 12 months after revascularization might represent an appropriate time for cardiac function reassessment.

## 5 LIMITATIONS

Our study has several limitations. The study was based on a single center, and therefore its generalizability may be limited. Not all patients had EF reassessment during follow‐up or at any time interval, which may induce selection biases inevitably. In this study, only echocardiography measurements in the same hospital were used to compare serial measurements of the EF since institutions and specific methods for measuring EF may vary. Of 3924 person‐time EF measurements, 1543 (97.1%) EF measurements before revascularization and 2202 (94.3%) EF measurements during follow‐up were by Simpson, respectively. Patients who had EF measurements by hospitals other than the index hospital (Anzhen) echocardiography were excluded. This restriction improved the accuracy of the EF measurements, but increased the number of patients who were lost to follow‐up. Studies that have used retrospective death certificate‐based methodology to identify cases of SCD are likely to overestimate the SCD mortality rate.^27^ Only 13 patients had ICD therapy during the follow‐up. But the data on ICD shocks or aborted sudden cardiac arrest were lacking. However, it was unlikely to have affected current findings because of their uses were minimal in this study population.

## 6 CONCLUSION

EF changed over time after revascularization in patients with LV dysfunction. When EF was reassessed early (<3 months) after revascularization, EF of 35% or less did not discriminate against the risk of SCD and had no influence in the mode of death as it did when EF was reassessed later (3–12 months). Our data suggested that 3–12 months after revascularization may be more appropriate for cardiac function reassessment than the usual reassessment at <3 months. A prospective study should be performed to determine what is the optimal time of EF reassessment to optimize ICD utilization.

## CONFLICT OF INTEREST STATEMENT

The authors declare no conflict of interest.

## Supporting information

Supporting information.

## Data Availability

The data generated or analyzed during this study are available from the corresponding author on reasonable requests.
