# Supplementary material for: Appropriate time for ejection fraction reassessment after revascularization in patients with left ventricular dysfunction for risk stratification of sudden cardiac death
Source: Clin Cardiol. 2023 Nov 7;47(1):e24162. doi: 10.1002/clc.24162 (PMC10766128; doi:10.1002/clc.24162)
Supplement: Supplementary file 1 — Supporting information. [file CLC-47-e24162-s001.docx]

Table 1. Factors Associated with all-cause death and SCD among patients who had EF reassessed <3 months after revascularization

| **Variables** | **All-cause death** | | | | **SCD** | | | |
| --- | --- | --- | --- | --- | --- | --- | --- | --- |
|  | Univariable analysis | | Multivariable analysis | | Univariable analysis | | Multivariable analysis | |
|  | HR(95%CI) | P value | HR(95%CI) | P value | HR(95%CI) | P value | HR(95%CI) | P value |
| Age | 1.03(1.02-1.05) | <0.001 | 1.03(1.01-1.05) | <0.001 | 1.01(0.98-1.03) | 0.510 |  |  |
| Male sex | 1.06(0.71-1.60) | 0.768 |  |  | 0.86(0.44-1.66) | 0.647 |  |  |
| Weight | 0.99(0.98-1.00) | 0.184 |  |  | 0.99(0.97-1.02) | 0.542 |  |  |
| Current smoking | 0.97(0.71-1.32) | 0.835 |  |  | 0.90(0.53-1.52) | 0.690 |  |  |
| Hypertension | 1.57(1.17-2.12) | 0.003 | 1.35(0.98-1.85) | 0.063 | 1.73(1.04-2.89) | 0.036 | 1.57(0.93-2.66) | 0.091 |
| Diabetes | 1.35(0.77-2.38) | 0.298 |  |  | 1.10(0.39-3.13) | 0.860 |  |  |
| eGFR | 0.99(0.98-1.00) | 0.002 |  |  | 0.99(0.98-1.00) | 0.049 |  |  |
| Cerebral vascular disease | 1.42(0.89-2.26) | 0.139 |  |  | 1.13(0.49-2.60) | 0.780 |  |  |
| History of MI | 0.89(0.66-1.19) | 0.437 |  |  | 0.95(0.58-1.55) | 0.824 |  |  |
| History of anterior MI | 1.14(0.78-1.66) | 0.501 |  |  | 1.35(0.74-2.44) | 0.326 |  |  |
| Bundle branch block | 1.24(0.71-2.18) | 0.455 |  |  | 0.50(0.13-1.94) | 0.315 |  |  |
| Initial EF | 0.97(0.94-1.00) | 0.057 |  |  | 0.97(0.93-1.02) | 0.201 |  |  |
| Reassessed (<3 months)  EF≤35% | 1.61(1.19-2.19) | 0.002 | 1.67(1.22-2.29) | 0.002 | 1.60(0.96-2.68) | 0.072 | 1.44(0.84-2.48) | 0.181 |
| ACS | 0.83(0.62-1.12) | 0.231 |  |  | 0.65(0.40-1.07) | 0.093 | 0.63(0.37-1.06) | 0.079 |
| Multivessel disease | 1.76(1.00-3.11) | 0.049 | 1.80(0.94-3.43) | 0.076 | 1.21(0.52-2.83) | 0.664 |  |  |
| Left main disease | 1.51(0.98-2.35) | 0.064 | 1.47(0.93-2.31) | 0.100 | 1.21(0.56-2.63) | 0.632 |  |  |
| PCI* | 0.78(0.53-1.15) | 0.207 |  |  | 0.78(0.41-1.48) | 0.445 |  |  |
| Complete revascularization | 1.09(0.80-1.48) | 0.595 |  |  | 1.19(0.71-2.00) | 0.504 |  |  |
| Beta blockers | 0.78(0.56-1.09) | 0.150 |  |  | 1.24(0.66-2.32) | 0.498 |  |  |
| ACEI/ARB | 0.95(0.70-1.30) | 0.767 |  |  | 1.20(0.73-1.98) | 0.480 |  |  |
| Aldosterone blockers | 0.92(0.56-1.52) | 0.742 |  |  | 1.26(0.60-2.64) | 0.537 |  |  |

*CABG was set as reference to PCI. Abbreviations: SCD, sudden cardiac death; HR, hazard ratio; CI, confidence interval; eGFR, estimated glomerular filtration rate; MI, myocardial infarction; EF, ejection fraction; ACS, acute coronary syndrome; PCI, percutaneous coronary intervention; CABG, coronary artery bypass grafting; ACEI, angiotensin-converting enzyme inhibitor; ARB, angiotensin receptor blocker.

Table 2. Factors Associated with all-cause death and SCD among patients who had EF reassessed 3-12 months after revascularization

| **Variables** | **All-cause death** | | | | **SCD** | | | |
| --- | --- | --- | --- | --- | --- | --- | --- | --- |
|  | Univariable analysis | | Multivariable analysis | | Univariable analysis | | Multivariable analysis | |
|  | HR(95%CI) | P value | HR(95%CI) | P value | HR(95%CI) | P value | HR(95%CI) | P value |
| Age | 1.04(1.01-1.06) | 0.002 |  |  | 1.02(0.98-1.06) | 0.265 |  |  |
| Male sex | 0.71(0.40-1.25) | 0.234 |  |  | 0.62(0.26-1.47) | 0.278 |  |  |
| Weight | 0.98(0.96-1.00) | 0.106 |  |  | 0.97(0.93-1.00) | 0.063 | 0.97(0.94-1.00) | 0.061 |
| Current smoking | 0.79(0.49-1.29) | 0.345 |  |  | 0.65(0.30-1.42) | 0.281 |  |  |
| Hypertension | 1.48(0.91-2.39) | 0.114 |  |  | 1.81(0.84-3.91) | 0.128 |  |  |
| Diabetes | 1.67(0.89-3.12) | 0.108 |  |  | 1.24(0.41-3.71) | 0.703 |  |  |
| eGFR | 0.98(0.97-0.99) | <0.001 | 0.98(0.97-0.99) | <0.001 | 0.98(0.96-1.00) | 0.027 | 0.98(0.96-1.00) | 0.055 |
| Cerebral vascular disease | 0.99(0.48-2.08) | 0.988 |  |  | 0.94(0.29-3.01) | 0.911 |  |  |
| History of MI | 1.29(0.82-2.03) | 0.277 |  |  | 0.88(0.44-1.78) | 0.724 |  |  |
| History of anterior MI | 1.11(0.63-1.96) | 0.729 |  |  | 0.67(0.24-1.87) | 0.445 |  |  |
| Bundle branch block | 1.34(0.54-3.33) | 0.527 |  |  | 1.32(0.30-5.84) | 0.712 |  |  |
| Initial EF | 1.00(0.95-1.05) | 0.972 |  |  | 0.95(0.89-1.01) | 0.116 |  |  |
| Reassessed (3-12 months)  EF≤35% | 1.85(1.12-3.05) | 0.017 | 1.81(1.06-3.10) | 0.029 | 2.74(1.33-5.62) | 0.006 | 2.71(1.31-5.61) | 0.007 |
| ACS | 0.92(0.57-1.49) | 0.741 |  |  | 0.81(0.39-1.66) | 0.560 |  |  |
| Multivessel disease | 0.62(0.36-1.08) | 0.049 |  |  | 0.56(0.25-1.25) | 0.159 |  |  |
| Left main disease | 0.68(0.25-1.88) | 0.460 |  |  | 0.44(0.06-3.32) | 0.427 |  |  |
| PCI* | 1.19(0.74-1.91) | 0.463 |  |  | 1.01(0.50-2.06) | 0.981 |  |  |
| Complete revascularization | 0.82(0.52-1.30) | 0.409 |  |  | 0.75(0.37-1.52) | 0.424 |  |  |
| Beta blockers | 0.61(0.37-1.00) | 0.049 | 0.60(0.36-1.02) | 0.061 | 0.77(0.34-1.75) | 0.535 |  |  |
| ACEI/ARB | 1.10(0.69-1.75) | 0.682 |  |  | 0.90(0.44-1.84) | 0.766 |  |  |
| Aldosterone blockers | 1.49(0.84-2.64) | 0.172 |  |  | 1.52(0.66-3.52) | 0.326 |  |  |

*CABG was set as reference to PCI. Abbreviations: SCD, sudden cardiac death; HR, hazard ratio; CI, confidence interval; eGFR, estimated glomerular filtration rate; MI, myocardial infarction; EF, ejection fraction; ACS, acute coronary syndrome; PCI, percutaneous coronary intervention; CABG, coronary artery bypass grafting; ACEI, angiotensin-converting enzyme inhibitor; ARB, angiotensin receptor blocker.
